# Cognitive Empathy and the Dark Triad: A Literature Review

**DOI:** 10.3390/ejihpe13110184

**Published:** 2023-11-13

**Authors:** Mirko Duradoni, Mustafa Can Gursesli, Maria Fiorenza, Alessia Donati, Andrea Guazzini

**Affiliations:** 1 Department of Education, Literatures, Intercultural Studies, Languages and Psychology, University of Florence, 50135 Florence, Italy; mustafacan.gursesli@unifi.it (M.C.G.); maria.fiorenza1@unifi.it (M.F.); andrea.guazzini@unifi.it (A.G.); 2 Department of Information Engineering, University of Florence, 50139 Florence, Italy; 3 Centre for the Study of Complex Dynamics, University of Florence, 50019 Florence, Italy

**Keywords:** Dark Triad, cognitive empathy, narcissism, Machiavellianism, psychopathy, empathy

## Abstract

This literature review aims to analyze studies published by researchers on the topic of the relationship between the psychological constructs of the Dark Triad and Cognitive Empathy. This study hypothesizes how having good cognitive empathic skills could benefit people who demonstrate Dark Triad traits, as this could facilitate the implementation of manipulative strategies. Through the process of identifying studies via databases and registers, 23 studies were included in this literature review, and the results and theories brought forward by the researchers find more agreement regarding the individual components of the Dark Triad than the whole construct: narcissism seems to have, for the most part, relatively small and typical positive correlations (more than 50% of correlations), Machiavellianism has relatively small and typical negative relationships (about 80% of correlations), and psychopathy has relatively large negative relationships (about 90% of correlations). This study conveys that Machiavellians and psychopaths, having reduced empathic abilities, use manipulation techniques that do not have to do with empathy (for example seduction, intimidation etc.), while narcissists would be, among these three dimensions, those most likely to understand others’ states of mind and thus be able to use this knowledge to their advantage—although there are doubts about the veracity of the statements and answers given by narcissists in the tests administered to them. This literature review could be a valid aid to professionals dealing with people who exhibit Dark Triad traits; understanding how those exhibiting Dark Triad traits manage their empathic abilities, the areas in which the various dimensions show deficits or not, and how they act to implement their manipulative and controlling tactics could aid in the development of more effective helping strategies to be utilized in therapy settings.

## 1. Introduction

### 1.1. The Dark Triad

In recent years there has been an exponential surge of interest [1] towards the understanding of the darker side of human nature, and in particular to the traits involved in the Dark Triad, a psychological construct that describes three remarkably abusive but not pathological personality types: Machiavellianism, subclinical narcissism, and subclinical psychopathy, which have been found to be highly correlated with one another. These three traits, defined precisely as “dark” because of their malevolent orientation and a so-called “callous” and manipulative social tendency, are conceptually distinct, despite the fact that they are very often found in correlation with each other. Regarding the clinical significance of this construct, as already written above, when we speak of the Dark Triad we mean the set of traits already described that manifest themselves in a non-severe dimension, as far as narcissism and psychopathy are concerned; Machiavellianism, for its part, cannot have clinical or subclinical dimensions given that it is referred to in the scientific literature as personality trait, and it has never been considered a disorder, nor has it been referenced in any version of the Diagnostic and Statistical Manual of Mental Disorders, so it is treated as strictly a personality construct. The Dark Triad, therefore, is not itself a clinical construct.

Regarding the history of this psychological construct, the article published by McHoskey, Worzel, and Szyarto in 1998 [2] was the first that examined the relationships between Machiavellianism, psychopathy, and narcissism, stating that, in nonclinical samples, these three constructs were not only highly correlated, but were almost overlapping.

With these findings and statements, these three researchers mobilized the scientific world to research the issue of the relationship between these constructs, and four years later, in 2002, the first scientific publication was made naming the strong link between narcissism, Machiavellianism, and psychopathy, by Delroy L. Paulhus and Kevin M. Williams. In the research, it was found how, due to cognitive, behavioral, and personality differences, the three traits examined are indeed distinguishable from each other, but nonetheless significantly related.

As stated above, each of these personality types is termed “dark” because they are believed to contain malevolent qualities, and in fact these traits are often associated with ethical, moral, and socially deviant behavior [3]. Narcissism is a personality style that manifests itself through exaggerated feelings of self-centeredness, which justify and entitle the narcissist to feel proud and superior over others and display attitudes of dominance and exploitation toward those around him or her; this haughty sense of self is nurtured by the narcissistic person’s exaggerated need to be appreciated and admired by others. The causes behind the development of narcissism are unclear at this time, but there is generally talk of a genetic predisposition interacting with an environment that fosters the emergence of the sets of behaviors that characterize narcissism [4]. Regarding the unity of this disorder, although the diagnostic manuals do not consider narcissism to be a disorder with multiple declinations, over the years many scholars and researchers have provided various different profiles that fall under the framework of narcissism. In general, two declinations of the construct in particular are accepted in the literature, namely, that of grandiose or overt narcissism and that of vulnerable or covert narcissism: the former presents exaggerated self-esteem and strong feelings of importance, while the latter is more often associated with defensive conduct and fragility, a high sensitivity to criticism, and a withdrawal from social life [5].

Psychopathy is characterized by persistent antisocial behavior, severe deficits in the area of empathy, an absence of remorse or guilt, and proud, uninhibited, and selfish personality traits [6]. The tendency to conduct amoral, violent, and cruel conduct is also present, as well as to have perverted attitudes and an unhealthy obsession with one’s self, in addition to demonstrating manipulative behaviors in interpersonal relationships; furthermore, psychopaths are very impulsive people and often very strong sensation seekers [7]. In the Dark Triad framework, psychopathy is certainly considered the “darkest” element, given the great correlation it has with exhibiting criminal and violent conduct [8]. Genetic elements (especially regarding the subtype of primary psychopathy, also known as “biological” psychopathy), and elements regarding the environment in which the psychopath lives (especially regarding the subtype of secondary psychopathy, also known as “learned” psychopathy), are counted among the causes of this condition. As previously mentioned, there are many theories on psychopathy that talk about its facets and subtypes, but in general, two subtypes are recognized, namely, primary and secondary psychopathy. Although these are phenotypically similar, they differ in when and how they manifest; while primary psychopathy arises from a genetic predisposition, which leads the person to have real deficits in the area of empathy, etc., secondary psychopathy, on the other hand, arises from a development in an environment that may predispose to it through the potential traumatic nature of certain events or situations that are experienced [9,10,11,12].

In contrast to narcissism and psychopathy, which are personality disorders marked in diagnostic manuals as psychopathologies, Machiavellianism is considered in the literature to be a personality trait, characterized by strong amorality, a deceptive and cynical temperament, callousness and indifference to ethical conduct, and indeed the use of manipulative techniques directed toward others, the exploitation of which is used by Machiavellians to achieve their goals [13]. Despite the fact that it is not recognized as a true disorder but more as a social orientation, scholars have nonetheless searched for its origins in both the genetic predispositions of those with this trait and the environment in which their development occurred, and results have been found in both areas, showing stronger correlations than psychopathy and narcissism with regard to the shared environment (for example, when one is together with peer groups or siblings [14]). The genetic origins of this social orientation are, however, recognized by researchers, especially considering the psychopathologies with which this trait correlates most, such as primary psychopathy (i.e., the “biological” subtype), that have as the basis of their origin precisely such inclinations [15].

As illustrated in this summary, there are overlapping characteristics shared among these traits, though debate remains about the presence of a common core [16].

Despite this ongoing discussion, it is becoming more widely accepted that there is a shared exploitative demeanor across the three traits, enabling the goal-focused manipulation of others’ emotions. Nagler and colleagues, in 2014, found that individuals high in Dark Triad traits are emotionally manipulative and prone to engage in callous exploitation—even if it seems that every trait per se manipulates others following patterns different from one another and to achieve different goals [17]. This characteristic, shared by the three traits, has made its way into the already frequent research regarding the Dark Triad, given that it is one of the most damaging interpersonal outcomes of this set of traits. The question regarding the origins of this mechanism and the emotional competence of people that display the Dark Triad traits has been the subject of numerous studies—despite the results obtained that have been described as mixed and unclear. Underlying the manipulation mechanisms already mentioned may be the construct of empathy.

### 1.2. Cognitive Empathy

Empathy is a complex phenomenon that has attracted the interest of psychologists from different fields (e.g., clinical, developmental, personality, social), as well as a wide range of scholars, including anthropologists, philosophers, and theologians [18]. Human beings can empathize with others and thus share their feelings and emotions in the absence of direct emotional stimulation [19].

The ability to “understand”, “perceive”, and “comprehend” the mental and affective states of our fellow animals is a crucial point of our existence as “social animals”. It enables us not only to communicate and interact with others effectively and pleasantly, but also to predict the actions, intentions, and feelings of others [20]. This ability to share involves a greater level of understanding of the mental states and actions, present and future, of the people around us, and may promote prosocial behavior [20].

Initially, the empathic construct placed more emphasis on deliberate cognitive processes, thus stressing the role of the empathic phenomenon in increasing social acuity, (i.e., the ability to accurately perceive the internal states of others) [21,22,23,24]. In the second half of the 20th century, however, most scientists adopted an “affective” perspective (i.e., the emotional component of empathy), arguing that a prerequisite for an empathic response was an understanding of the deeper inner feelings and states of others [25,26,27]. Thereafter, the construct was then approached from a multidimensional perspective, focusing on the dual nature of the phenomenon [28,29,30,31,32,33]. Within this context, the contribution of the psychologist [28] was one of the most consistent attempts to address the complexity of the empathy construct, in support of an integrated approach that recognizes the role of cognitions and affections and highlights the interaction between the different expressions of empathic responsiveness.

The dual nature of empathic responses is represented by two closely related but distinct processes: an affective one, which refers to the experience of emotionally sharing another person’s state of mind [34]—comparable to the construct of emotional contagion [35,36]; and a cognitive one, which refers to the ability to recognize and understand another person’s thoughts, intentions and feelings [37,38]. Neuroscientific evidence supports the existence of two possible systems involved in empathic expression, one affective and the other cognitive, capable of activating brain networks that are separate but interact with each other. In the development of a cognitive empathic response, the neural circuits typically involved are those of the cognitive and affective theory of mind [39].

Cognitive empathy, since it provides sensitive emotional information, may also underlie manipulative personalities [40] and can become a valuable tool in attempts at exploration of the phenomenon. In the meta-analysis done by Miao and colleagues [41], where the Dark Triad is associated with Emotional Intelligence, a construct which represents “a constellation of behavioral dispositions concerning one’s ability to recognize, process, and utilize emotion-laden information” [42] and that could be strictly involved with Cognitive Empathy, authors explain how some researchers are concerned that high EI could give people the ability to take advantage of others by manipulating their emotions. Some scholars have argued that EI could be associated with antisocial impulsive features, managing others’ emotions to achieve personal goals, ingratiating supervisors by reporting successes and hiding failures, and mortifying others to maximize personal gains [17,43,44]—but it is made clear in this meta-analysis that the majority of EI researchers, however, state that EI is related to empathy and positive, prosocial behavior.

Although research has demonstrated robust negative relationships between the dark personalities and empathy, findings are inherently limited [45]. Consequently, the identified empathic deficits associated with the Dark Triad cannot be reliably acknowledged as either being cognitive or affective. This information is extremely important, given the distinct behavioral and motivational attributes of the two empathy systems.

### 1.3. Theoretical Assumptions of the Relationship between the Constructs Examined

On the relationship between the Dark Triad and cognitive empathy, the existing literature numbers many different positions based on the results of previous studies performed on the subject, which have generated theories on the nature of the relationship between these two constructs. Some of the theoretical assumptions made by researchers will be listed here to present schematically the many views on the subject and then we will proceed with our analysis of the literature.

In the research carried out by Wai and Tiliopoulos [45] in 2012, which was the first study in which empathic deficits were examined in a two-dimensional key and toward all three components of the Dark Triad, it is expressed that the definition of cognitive empathy is the ability to discriminate the moods of others without, however, being affected by those emotions; “this, as a skill”, the authors continue, “provides an individual with the ability to understand important information about the feelings of others, it can also be of great help to those personalities who exhibit manipulative traits” [40].

Instead, taking up the article by Jonason and Kroll [46], published in 2015, the authors break down the Dark Triad into its three dimensions, surmising how, for those who score high on narcissism, it may be advantageous to have equally high scores on empathy—being empathetic, in fact, may facilitate access to the approval of others, which narcissists need [47]. The study then goes on to point out how, according to an evolutionary approach, having limited empathy might help facilitate an active exploitation of others [1,48]; in order to use another person, one does not have to empathize with said person. This is reflected in the mechanisms and behaviors of psychopathic people, engaging in a brutality and violence not found in the manipulative tactics of narcissists [49]. In the recent research, published in 2019 by Turner [50] and colleagues, however, they express a contrary hypothesis to those in the two studies cited previously in this paragraph, namely, they believe that the Dark Triad traits correlate negatively with both affective and cognitive empathy since “negative links (vs. positive links) have been found more commonly in the literature” [50]. Indeed, in this study it is pointed out that previous research has shown that people characterized by the Dark Triad possess lower levels of cognitive empathy than the norm and that emotional deficits are shared by all three of its components [1,51].

Finally, in the 2019 research developed by Heym [52] and colleagues, it was recognized that a cognitive empathy ability is essential to assume the perspectives of others and for predicting another’s behavior, thus making manipulation possible and easier [53]. Further explored was how all Dark Triad traits are associated with reduced cognitive empathy, although this was determined only through psychopathy scores once shared variance was accounted for [48]. “In other cases”, the authors continue, “cognitive empathy does not appear to be a source of deficit for the Dark Triad, and in the particular case of narcissism it even appears to exceed thresholds considered normotypical; this could mean that narcissists possess better abilities in understanding the thoughts and intentions of others’’ [45].

### 1.4. Aim of the Review

This literature review aims to synthesize the available literature trailing the guidelines of the Preferred Reporting Items for Systematic Reviews and Meta-analysis (PRISMA) method, to methodically assess what the nature of the relationship between the cognitive empathy and Dark Triad constructs is, and to provide possible explanations presented by the authors of the research included here.

At last, a final note should be presented to the reader: this literature review investigates only the relationship between the Dark Triad and the cognitive part of empathy, rather than researching the empathy construct as a whole or searching the affective side of empathy too. This was a choice made by the authors that follows the literature findings and results regarding the possible affiliation between the Dark Triad traits and cognitive empathy: in the literature review by Van Langen and colleagues [54], it was found that cognitive empathy is more strongly associated with offending (d = 0.43) than affective empathy (d = 0.19), while according to Smith [53], it may be involved in antisocial behavior [55] since, by increasing social functioning [56,57,58], it makes one capable of understanding and predicting others’ behaviors, thus giving one the ability to manipulate or deceive others for personal gain [53]. The 2017 study by van Zonneveld [59] and colleagues found how, in a sample of children considered to be at high risk for engaging in criminal behavior in the future, cognitive empathy is intact at the expense of affective empathy.

Given this evidence from the literature, it is assumed that people with the Dark Triad traits possess a deficit in the affective side of empathy, however, this does not result in other limitations with regard to the cognitive side of empathy; therefore, cognitive empathic skills can be used by these people for the purposes of obtaining the environmental and personal information suitable for manipulating others. Thus, exploring the link between the Dark Triad and cognitive empathy in more depth may provide new and useful information for enrichment of the literature. This literature review can have an impact on cultural and social aspects, contributing to people’s lives and, more broadly, to society as a whole, which goes beyond academic research. In particular, it could be helpful to professionals working in the broad field of psychology, especially for those dealing with people who exhibit Dark Triad traits; understanding how they manage their empathic abilities, what the areas are in which the various dimensions show deficits or not, and how they manage to implement their manipulative and controlling tactics could aid in the development of more effective helping strategies to assist them as they go through their psychological journey—which can take place either in the setting of a private practice or in the public setting of a hospital or even in Therapeutic Rehabilitation Communities.

## 2. Materials and Methods

### 2.1. Research Strategy

#### Methods, Procedures, Synthesis and Screening Process

Information sources: this literature review was conducted following the guidelines of the Preferred Reporting Items for Systematic Reviews and Meta-analysis (PRISMA). Following these instructions, scientific studies on the relationship between the personality pattern referred to as the Dark Triad and the cognitive empathy construct were searched for, using the keywords “dark triad” and “cognitive empathy”.

Literature research, data collection processes and results of the study selection through inclusion and exclusion criteria: through the consultation of Google Scholar, PsycInfo, PsycArticles, PubMed, Science Direct, Sociological Abstracts, and Academic Search Complete databases with the keywords “dark triad” and “cognitive empathy”, 1107 indexed results were obtained, of which 1077 abstracts were examined; the 30 abstracts that were not screened were so because (i) they were duplicates of other articles (12), (ii) they could not be accessed (9), (iii) they showed up in scientific search engines as just citations (4), and (iv) they were reported in languages other than Italian and English (5). From the abstracts that were consulted (1077), 225 were eliminated because they did not meet the inclusion criteria, namely, (i) being completed and published by September 2022, (ii) being accessible for consultation directly from the site where the viewing took place, through subscriptions belonging to the University of Florence, or by request to the corresponding authors, (iii) being written in English or Italian, and (iv) being empirical studies.

**Figure 1:**
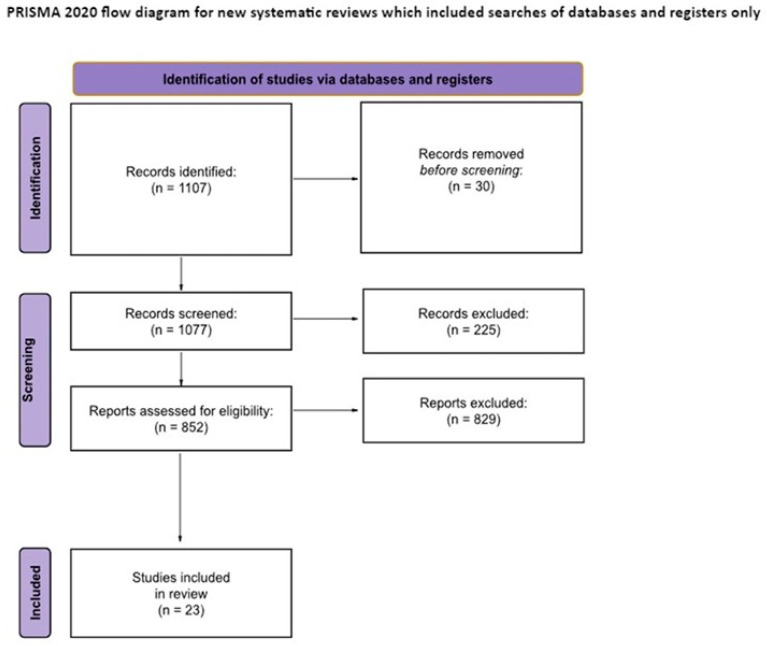
Diagram showing the flow of information through the review: the number of abstracts and articles identified, included, and excluded.

Subsequently, 852 full texts were reviewed, and of these 829 were excluded on the basis of exclusion criteria such as (i) not reporting appropriate measures required for the evaluation of the two constructs and (ii) missing prime data analysis that is needed to the review process (for example: a lack of inferential statistics, absence of correlation coefficients for variables of interest, etc.). The remaining 23 studies were included in this literature review (see Figure 1).

The authors (M.D., M.F., and A.D.) screened titles and abstracts for possible inclusion and exclusion criteria; 225 abstracts were excluded according to these criteria. After this process, the authors (M.D., M.C.G., A.G., A.D., and M.F.) independently screened 852 articles and decided that 23 articles were eligible for inclusion. We clarify that the sources were not distributed among the reviewers and, therefore, the same set of sources was reviewed a total of five times.

Since this literature review aimed to assess the strength of the relationship between the Dark Triad and Cognitive Empathy, we considered the operationalization of the latter as an effect measure.

**Figure 2:**
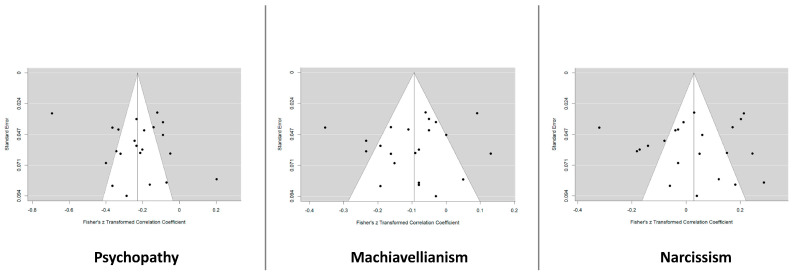
Funnel plot analysis of the included studies divided for each Dark Triad trait.

In order to synthesize these findings, a narrative approach was used to present the key findings of the selected studies in two tables (Appendix A and Appendix B). The collected data were examined by the three reviewers involved to identify common themes, patterns, and trends regarding the relationship between the Dark Triad and cognitive empathy. The risk of bias for each included study was assessed individually by the first reviewers, with any disagreements being resolved by the third reviewer (M.D.). This evaluation was conducted following the internal protocol guidelines derived by the STROBE Checklist for correlational studies to limit arbitrary judgments. We also assessed heterogeneity using the inconsistency index (I2) and Funnel plot analysis, Egger’s regression, and Kendalls Tau to assess the presence of publication bias. The included studies showed a very high heterogeneity (ranging from 81% to 91.5%) and no publication bias (see Figure 2 for funnel plots and Figure 2 for the studies eligible for heterogeneity analysis). A canonical sensitivity analysis and a certainty assessment, which are commonly used in intervention studies, were considered inappropriate for reviews of correlational and/or cross-sectional studies, as they do not aim to estimate a specific value but focus on the presentation of a distribution. The methodological quality and potential bias of the included studies were assessed by considering factors such as study design, sample size, data collection methods, statistical analyses and reporting quality, to provide a comprehensive assessment of the available evidence.

### 2.2. Description of Included Studies

At the end of the selection process, 23 empirical studies were included, with all of them being cross-sectional and statistically analyzing the relationship between the Dark Triad construct and the cognitive empathy construct.

#### 2.2.1. Country of Publication of the Included Studies

The research included in this literature review follows a geographic distribution with a majority of studies published in countries considered Western, the first of which included European countries such as Italy, Serbia, Croatia, Germany, Sweden, and the United Kingdom (a total of 10 studies thus distributed: 3 studies were published in Serbia, Italy and Croatia score 2 studies each, and the other three count one study per country). This is followed by the United States, with 7 studies, making it the country with the most studies published in total, and right behind is Australia and Canada (with a total of 4 studies, 3 published in Australia and the remaining one in Canada). Both Russia and China have only one study published in their respective country, and of these two, China is the only Asian country to have published a study investigating the relationship between the Dark Triad and cognitive empathy.

It should be brought to consideration that the distribution of countries just described reflects the fact that papers not written in English or Italian were excluded, as stated in the exclusion criteria at the beginning of this chapter.

#### 2.2.2. Years of Publication of the Included Studies

In the decade spanning from 2012 to 2022, all 23 studies included in this literature review were published, and we can see a fairly growing interest in the relationship between the two constructs. Demonstrating this, thirteen studies were published before 2020 (2012, 2013, and 2015 counting one study each, in 2017 and 2019 there were 2 studies published for each year, and in 2016 and 2018 three studies per year), and after that year up until 2022, there were as many as ten published studies (three in 2020, six in 2021, and one in 2022).

#### 2.2.3. Mean Ages of the Samples in the Included Studies

The mean age of participants in the studies that were included in this review follows a peculiar distribution, as only one of them has a mean age above forty years in one of the two samples considered [60]. In fact, the rest of the studies are composed of samples mainly consisting of college students: there are 12 studies whose sample exhibits an average age between twenty and thirty years old, another 4 have an average age between thirty and thirty-five years old, and only 2 have an average between thirty-five and forty years old. Lastly, as many as 4 studies exhibit an average age of less than twenty years.

#### 2.2.4. Gender Distributions of the Samples in the Included Studies

The distribution of sexes in the samples (indicated here, for convenience, by the percentage of females in each study) follows a trend with a majority of 21 studies in which women and girls exceed 50% of the participants, and one of the studies even has an all-female sample. Only two of the studies have a female percentage in their sample that is lower than 50%, and the others have female percentages distributed like so: in between 50% and 60% seven studies fall, in the interval from 60% to 70% we find four studies, from 70% to 80% there are eight studies, and finally from 80% to 85% we find two studies.

Only one of the studies [60] presented here also took care to indicate gender-neutral participants (18, in a sample of 532 participants, with a percentage of cisgender females at 53.20%), i.e., people who do not reflect and feel at ease in the cultural conceptions of man and woman typical of modern societies; for the other studies, it was not indicated whether only the sex assigned at birth component was considered in the participant selection process to indicate the division between males and females or whether the person’s perceived gender alignment was also considered.

#### 2.2.5. Instruments Used in the Included Studies to Measure the Two Constructs

##### Dark Triad

In terms of the instruments chosen for measuring the Dark Triad, as many as 13 studies use the Short Dark Triad (SD3) developed by Jones & Paulhus in 2014 [61] in their research, while another 8 use the Dark Triad Dirty Dozen (DTDD) created by Jonason & Webster in 2010 [62]; these, in fact, are the two best-known short measures (the first consists of 27 items and the second of 12) created specifically for Dark Triad assessment. Of these 21 total studies, one uses both tests on the chosen sample and three others use other supplementary measures to measure one or more of the specific components of the Dark Triad. Only two studies used three different instruments to assess each dimension of this construct.

**Figure 3:**
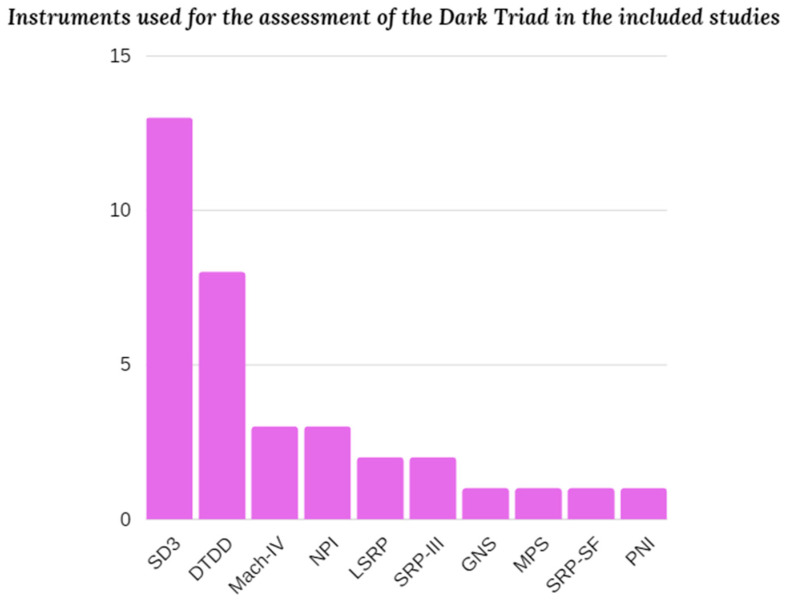
Bar graph showing the instruments used to measure the Dark Triad in the various studies included.

The tests used that are not the questionnaires mentioned above are as follows: the Mach-IV [63], the Narcissistic personality inventory (NPI; [64]), the Self-Report Psychopathy Scale (SRP-III; [65]), the Levenson self-report psychopathy scale (LSRP; [66]), the Grandiose Narcissism Scale (GNS; [67]), the Machiavellianism Personality Scale (MPS; [68]), the Self-Report Psychopathy Scale—Short Form (SRP-SF; [69]), and the Pathological Narcissism Inventory (PNI; [70]) (See Figure 3).

##### Cognitive Empathy

**Figure 4:**
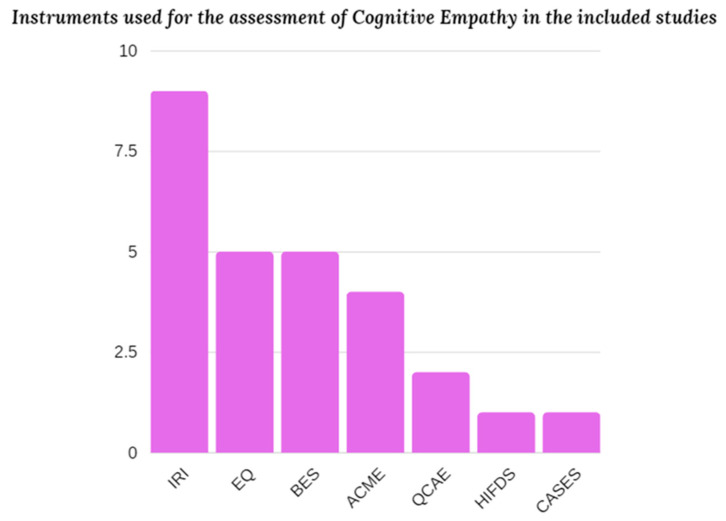
Bar graph showing the instruments used to measure cognitive empathy in the various studies included.

Unlike the instruments used for Dark Triad assessment, there are many more specific tests that serve the function of measuring empathy, most of which were developed to assess both the affective and cognitive components of empathy, and in fact the measures used by the included studies have some scales built specifically to measure cognitive empathy: for example, The Interpersonal Reactivity Index [71], used by as many as 9 studies, includes a scale called “Perspective Taking” that examines precisely the cognitive component of empathy; the same is true for the Empathy Quotient (EQ; [72]), used by 5 studies, and the Basic Empathy Scale [73], used by as many studies. The Affective and Cognitive Measure of Empathy (ACME; [74]) is, among the measures used by the included studies (4 to be precise), the most recent, followed next by the Questionnaire of Cognitive and Affective Empathy [75], which was employed by 2 studies. Finally, there were 4 studies in which more than one questionnaire and/or test for cognitive empathy was used for measurement. The tests used that are not the questionnaires indicated above are the following: the How I Feel in Different Situations Scale [29,76], and The Cognitive, Affective, and Somatic Empathy Scales (CASES; [77]) (See Figure 4).

##### Uni-Dimensionality and Multidimensionality of the Instruments by Which Studies Have Measured the Dark Triad and Cognitive Empathy

A particular feature of the tests used by the research included in this literature review is that, for both those that measure the Dark Triad and those that measure cognitive empathy instead, all of them are multidimensional—meaning these are tests that are divided into scales and/or subscales that each measure a particular and specific area of the construct on which the instrument is based.

One note to be presented in this digression is at the level of the instruments used to measure cognitive empathy: one of the studies [78] included here uses a measure, called the “Reading the Mind in the Eyes Test” (RMET; [72]), which is one-dimensional; this measure was reported (Appendix A—Table A1) but not included among the tests that measure Cognitive Empathy because it is used as a practice test for assessing this trait in a person, but in truth it represents the construct of social intelligence, which is related to Cognitive Empathy—so it is an instrument that is placed alongside one that is based on measuring the Cognitive Empathy construct, but does not properly measure it itself.

## 3. Results

### 3.1. Main Results

The answer to the research question with which this review was initiated does not appear simple or obvious, finding varying results in the existing literature. While one of the key characteristics by which the Dark Triad is described is precisely the lack of empathy, the studies included here do find a clear negative correlation with this, but especially with regard to its affective component; in the case of the cognitive one, research does not seem to find a common answer, at least for the unitary construct. Much clearer results, which find agreement in almost all research, are found at the level of the individual components of the Dark Triad. Appendix A shows all the main features of the studies reviewed and the results obtained.

#### 3.1.1. Narcissism and Cognitive Empathy

The results of the studies included in this review dealing with the correlation between narcissism and cognitive empathy are mostly positive, counting as many as 21 correlations that, according to the guidelines of Gignac & Szodorai (2016) [79] range from low positive correlation (9), to medium positive correlation (9), and high positive correlation (3); but there are also six low negative correlations, four medium negative correlations, and three high negative correlations, with even a correlation coefficient as high as −0.31.

Although this review finds, for the most part, results in line with the literature, the 14 results that find negative relationships between the two variables are important to ask questions about the possible risks of biases assumed by the studies presented here, which may arise, for example, at the level of the instruments used.

**Figure 5:**
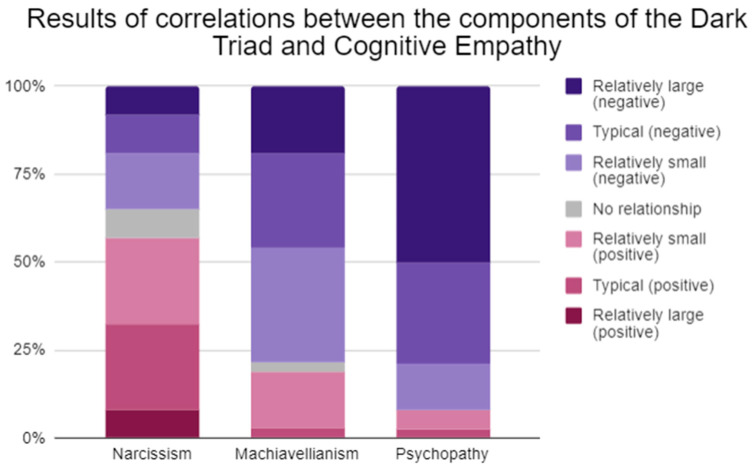
Stacked column chart comparing and summarizing the results of correlations between the components of the Dark Triad and cognitive empathy; Gignac & Szodorai’s [79] guidelines for interpreting correlation coefficients were used in this literature analysis.

Furthermore in most of the studies included here, narcissism is considered as a unitary disorder, not taking into consideration the subtypes of this that are instead outlined in the literature. Only one study in fact makes this distinction [80] and finds the results of the correlations between cognitive empathy and the two subtypes of narcissism—grandiose and vulnerable—are respectively −0.06 for the grandiose component of this construct and −0.17 for the vulnerable component (Figure 5).

#### 3.1.2. Machiavellianism and Cognitive Empathy

On a completely different tack, however, are the results of the correlations between cognitive empathy and Machiavellianism. With respect to narcissism, the negative direction of this relationship is clear, with as many as twelve low and negative correlations, ten medium and negative, five high and negative, and two exceeding the −0.30 threshold (−0.34 and −0.37). These results thus align with the literature’s view of a “brighter” and a “darker” part of the Dark Triad—the latter being composed, as mentioned earlier, of Machiavellianism and psychopathy. In addition to this orientation, which mostly turns toward a negative correlation, there are six studies that find a low and positive relationship and one that finds it medium and positive (Figure 5).

#### 3.1.3. Psychopathy and Cognitive Empathy

Compared with narcissism and Machiavellianism, the results concerning psychopathy are certainly the clearest, fully matching the results found in the literature: as many as 35 negative correlations were found by the research included here, counting five low and negative correlations, eleven medium and negative correlations, nine high and negative correlations, and as many as ten that exceeded −0.30. The other three correlation coefficients, two of them being low and positive and the other medium and positive, turn out to be the only exceptions (with values of 0.06, 0.07, and 0.20, respectively) to a largely and significantly negative trend. The agreement of the majority of the included studies reflects the thinking of the scientific community, which considers psychopathy, among the components of the Dark Triad, to have the most deficits in the area of empathy.

As with narcissism, which has been divided into two subtypes, psychopathy also has two forms—the primary or “biological” form and the secondary or “learned” form—which are investigated distinctly by only one study among those included [40]: for the primary subtype, the correlation coefficient found by that research was −0.16 while that found for the secondary subtype was −0.10 (Figure 5).

#### 3.1.4. Dark Triad in General and Cognitive Empathy

In the results of correlations between the Dark Triad in general (referred to as “Dark Triad Total” in the studies) and cognitive empathy, the large number of studies that do not report such a correlation stands out (see Table 1). This is due, as stated at the beginning of the paragraph, to the nature of the construct, which itself is divided into three significantly related components that were grouped under the name Dark Triad by Paulhus and Williams in 2002 [51]. Thus, most studies preferred to calculate the correlation coefficient of the individual components rather than report a single measure. For those studies that do report it instead, it comes as an additional index to the individual measurements made for narcissism, Machiavellianism, and psychopathy. Only one study solely calculated the score for the Dark Triad in general [81], indicating its total and partial correlation score (−0.14 and 0.12, respectively). Another study [82] reports a low and negative correlation (amounting to −0.02), while two others report high and negative correlations (−0.22 and −0.24, [48,83]), and yet another study [84] results in a highly significant positive correlation (0.44). As mentioned earlier—and as can be seen from the reported results—there is no real agreement on the relationship between the Dark Triad construct and cognitive empathy, but more unified results are recognized at the level of individual dimensions.

### 3.2. Study Limitations and Risks of Biases

Among the limitations present in most studies, the imbalance between the sexes in the samples analyzed is one of the most common, and very often the female presence is largely greater than the male presence. This, especially in studies that find a positive correlation between the components of the Dark Triad and cognitive empathy, could represent a risk of bias, since it has been shown in the scientific literature that women, compared to men, possess a greater empathic capacity even in conditions where the latter is inhibited (e.g., [46]). Most studies, moreover, use samples drawn from specific contexts (such as universities, for example) and/or from countries that have a population that can be considered as WEIRD (i.e., Western, Educated, Industrialized, Rich, and Democratic; see [96]). While this may be a strong limitation in that only one cultural system is primarily analyzed, it is in keeping with the tools used by these studies, which are standardized and calibrated to the characteristics described above, and may not be suitable for inclusion in other systems and cultures (see [83]). Many of the studies included here, as mentioned earlier, chose the participants who will later make up the sample from limited settings such as universities, and this results in the majority of the samples analyzed being composed of people of a young age, often making the average age very low and the age range limited. It would be desirable to use older age samples as well, so that the relationship of these variables with age variation can be controlled for. Finally, if we discuss sampling techniques, there are many studies included that use non-probabilistic samples, resorting to volunteers [82], to external apparatuses that deal precisely with the collection of participants for this type of research (e.g., MTurk, i.e., Amazon Mechanical Turk in ([60]), or even resorting to other techniques, such as the Snowball Technique (“Avalanche Sampling”, in [78]). This clearly can generate a number of not insignificant biases, such as predisposing volunteers to perform the test, or being able to examine only part of the population of interest, thus making the results obtained non-generalizable. In addition—as a final possible limitation regarding the sampling method and sample per se—some studies present a sample consisting of a rather low number of participants.

Turning instead to the measures used by studies to assess the Dark Triad, it can be seen that most studies use two short measures created specifically for the assessment of this construct, namely the Short Dark Triad (SD3) developed by Jones & Paulhus in 2014 [61] and the Dark Triad Dirty Dozen (DTDD) [95] created by Jonason & Webster in 2010. Despite the wide use of these tests and their respective efficiency, both have some shortcomings clearly illustrated by Maples and colleagues’ study published in 2014 [97]: the scores obtained in the SD3 manifest stronger convergent validity and incremental validity regarding the construct from which it originates than the DTDD, but it appears that the SD3 delineates a profile of narcissism by focusing on its grandiosity aspects, not also capturing the vulnerable side of this component of the Dark Triad, while the DTDD more accurately determines both of these aspects.

Instead, the measures that are used to assess cognitive empathy are many—among the most widely used we have the Interpersonal Reactivity Index (IRI, [71]), the Empathy Quotient (EQ; [72]), and the Basic Empathy Scale [73]—but these, rather than individually assessing the various facets of empathy (e.g., cognitive and affective empathy), use scales specifically for each component in a single test. One of the studies of those included [91] notes how the most widely used questionnaire for assessing empathy, i.e., the IRI, might incorrectly measure the construct of cognitive empathy as “sympathy”, which has among its meanings that of “compassion”, in addition to that of “sympathy” proper, and the more correct one—with respect to the construct under consideration here—of “understanding”.

All of the tests employed in the measurement of both the Dark Triad and cognitive empathy, then, are multidimensional in nature, which generally means that the construct the test measures is better represented, given the accuracy in reporting the various aspects of it. But, a majority of items (open-ended/multiple-response questions, etc., to which the person to whom the test is administered responds) encapsulated in multiple scales and subscales can invite other types of bias within the analysis. To conclude this section based on the measures used, the majority of the questionnaires used by the research included here are of the self-report type; it is well known how this type of test can result in the manifestation of the social desirability bias, i.e., that if certain cautions are not reported (such as ensuring anonymity) people tend to respond following a moral pattern, wanting to present themselves in a way that is considered positive by their culture; or again, test-takers may decide to respond in a way that they think is right or better than another. One case of this, which has everything to do with this research and has been noted by several among the studies reported here (e.g., [87]), is that one explanation for the high ability in understanding others’ emotions and perspective-taking—and thus cognitive empathy—of narcissists is that these people tend to overestimate their own abilities. This becomes more evident when measures that serve as assessments of the Dark Triad and narcissism are paired with tasks in which narcissists have to prove their abilities, and these do not achieve high scores, but rather in the normal range, in contrast to the reported opinion of self in self-report questionnaires. The grandiose component of this personality trait could therefore be a significant bias when this type of measure is adopted in the measurement. Appendix B (Table A2) shows all the key findings, limitations, and risks of biases present in the studies included in this literature review.

## 4. Discussion and Conclusions

This literature review of the literature on the psychological constructs of the Dark Triad and cognitive empathy, carried out with inspiration from the guidelines of the Preferred Reporting Items for Systematic reviews and Meta-Analyses (PRISMA) method, collected the empirical studies carried out on the subject with the aim of understanding the relationship between them. Although the literature developed on the subject is relatively recent, research on the subject has been implemented over the years, giving results that have led scholars to provide increasingly precise theses and theories aimed at explaining these outcomes.

The included studies overall report a positive correlation, small to typical, between narcissism and cognitive empathy (more than 50%), a negative correlation, small to typical, between Machiavellianism and cognitive empathy (about 80%), and a negative correlation, typical to large, between psychopathy and cognitive empathy (about 90%).

Regarding specifically narcissism, researchers believe that the manipulation techniques that the people displaying this trait use are based precisely on their ability to understand others’ emotions, thus utilizing their cognitive empathic abilities to their advantage in their relationships with others [49], and it is surmised how it can be advantageous for a narcissist to have empathic abilities, since this can facilitate access to others’ approval, which narcissists need [47]. In addition, narcissists also turn out to have empathic abilities in the affective component, but especially if the narcissism is of the vulnerable or covert type, whereas it is for the grandiose or overt type that cognitive empathy is intact at the expense of affective empathy [98]. Because of the cognitive empathy ability, as mentioned above, overt narcissists are able to manipulate others by understanding their moods, and the absence of the affective component means that they do not feel remorse or guilt in harming those around them. It should be noted, however, that these differences between the two types of narcissism and their relationship to empathy need more investigation: only one of the studies included here, in fact, explores both of these aspects and reports two different results for narcissism [80]. Other than this, it should be noted that while there were 21 positive correlations, there were also 13 negative correlations between narcissism and cognitive empathy. This means that while most of the studies correlated these two constructs, granting sensible explanations for this correlation, there is still a notable part of the studies that does not find or finds a negative correlation between them, making this correlation the “weakest” compared to the Machiavellianism and the psychopathy correlations to cognitive empathy.

As for Machiavellianism, Doyle [87] hypothesizes that the inherent nature of the Machiavellian, which drives this person to use manipulative-type strategies to achieve their ends without concern for consequences, makes the Machiavellian completely unmotivated to interact with others in ways that are not in his or her best interest; this means that Machiavellians possess “normal” abilities in the area of cognitive empathy, but do not exhibit any internal “drive” that allows them to use these abilities. Thus, it is believed that those who exhibit Machiavellian behaviors prefer to implement strategies that do not draw on cognitive empathy skills or empathic skills in general. Despite the gap that Machiavellians present with regard to cognitive empathy, this is less pronounced than the deficiencies presented by people with psychopathy, and Jonason and Krause point this out in their study: “Psychopathy and Machiavellianism correlate with low levels of affective empathy as opposed to narcissism. This suggests that each of the traits is accompanied by unique emotional deficiencies, but psychopathy facilitates the greatest number of emotional deficiencies” [48].

Finally, with regard to psychopathy, the research included here reports the most homogeneous result among the three dimensions of the Dark Triad. As mentioned earlier, psychopathy is the Dark Triad trait with the most significant empathic and emotional shortcomings. Researchers believe that psychopaths use other techniques, such as intimidation and seduction, to manipulate others, as they cannot resort to strategies that rely on understanding others’ states of mind [49]. Doyle [87] reports in his study how the negative association of cognitive empathy with psychopathy is a finding that suggests that individuals with high psychopathy scores are unable to lie effectively [53,99], continuing as follows: “it could be argued that psychopaths may have earned a reputation as liars and manipulators because of the frequency with which they enact these behaviors. However, there is only mixed evidence to support the idea that psychopathy is associated with success in these endeavors [100,101,102]”.

The results found by this literature review could imply that manipulation in people characterized by these three traits is carried out using different types of information, making the narcissist the one that mostly uses cognitive empathic abilities and the psychopath and the Machiavellian the ones that use instead other types of knowledge, as seen in the results and in the interpretations brought forth by the research included here. The findings that have been achieved from this literature review are reflected in some of the theoretical models published by scholars regarding empathy as a psychological construct.

Picking up on the theme discussed earlier, about how people who exhibit Dark Triad traits demonstrate a deficit with regard to the affective and emotional side of empathy—as largely evidenced by existing research in the literature—the Dual Route Model of empathy (first discussed by Yu and Chou in 2018 [103]), which is grafted onto neurobiological foundations, describes how there are two different “paths” that empathic processes can take: the first, of the affective type, is an automatic, fast, specific and low-energy pathway—it could almost be called the more “natural” route of the two, since according to the researchers it is established very early in a child’s life and is maintained throughout life as the first empathic approach one has toward others. The second path, on the other hand, is a cognitive one, and is defined as complex, slow, iterative, energy-consuming, and requiring active and conscious effort; it develops in the person later in life and it is a difficult path to follow instinctively, since it requires a definite disposition of attention and time on the part of the person undertaking it. The fact that Dark Triad traits do not normatively dispose of the affective pathway, as set forth above, does not preclude them from being able to use the cognitive one, and the fact that it is an “effortful” pathway, that is, one that has a definite purpose which is “goal-oriented”, makes it possible for them to use their cognitive empathic capacity with the goal of manipulating the other person, of exploiting him or her in some way that is advantageous to them.

The empathy “demonstrated” by people displaying Dark Triad traits, not being of the affective type—or even of the “positive” type—could be referred to as “tactical empathy”, that is, a type of empathy whose basic motivations may be seduction, fraud, manipulation, or violent tendencies [104]. Bubandt and Willerslev, in 2015 [104], state how “while the renewed interest in empathy promises a fresh look at the conditions of possibility of sociality itself, we argue that this potential can only be realized if we abandon the implicit notion that empathy is always a moral virtue and instead embrace a broader approach that includes its darker side as well; that empathic identifications with others often do not have as their goal mutual understanding, altruism, consolation, intersubjective compassion, caring or social cohesion—goals conventionally considered the sine qua non of empathy. Instead, the empathic faculty is used for deceptive and ultimately violent purposes” [104,105].

Continuing to analyze other types of “Dark Empathy”, “empathic sadism” or “empathic cruelty” is another form of selfish empathy that, in its basic form, means that an empathic observer enjoys another person’s pain or suffering [106]. The negative feelings of the other person are translated by the empathizer into positive ones. It can manifest in various forms, such as taking pleasure in sad tragedies and movies [107], as a motivation for punishment [106], in sadistic acts, in schadenfreude, and in everyday behaviors such as bullying, shaming, and teasing. Sadistic empathy often includes the manipulation of others and can be understood as creating a situation for the other person with the goal of making his or her emotional response to that situation intelligible, and thus shareable. Psychopaths, for example, fall under the description of sadistic empathy [105,108].

The relevance of these implications for the literature and future research is significant: if we can distinguish that the three dimensions of the Dark Triad employ manipulation techniques that differ from each other, by using different kinds of information, we can possibly understand how the information each trait receives is processed and where the manipulation attempts come from in all of the three dimensions. Deepening our knowledge regarding this can be a valid aid in constructing new therapies for people that display this personality pattern.

Regarding perspectives in research on the relationship between the constructs investigated in this literature review, there are many aspects that have not been adequately explored in the studies included here and that, in the future, could be investigated more carefully to assess the effect they may have on the relationship between the variables.

Starting with the participants who took part in the studies, the three factors that were found most in the chosen samples are the imbalance between the sex proportions (in most studies, the number of females far exceeded that of males), the very often low mean age, and the limited age range. It would be desirable for future studies to aim to make the samples more heterogeneous in these respects, since, as mentioned before, it has been shown in the scientific literature that females possess greater empathic abilities even in conditions where the latter are inhibited [46]; regarding the age, on the other hand, one could explore how the empathic capabilities of the Dark Triad vary depending on the person’s years.

These two components, however, are almost always related to the places where researchers choose to look for their sample—very often, in fact, they are universities, whose attendees are often young and highly educated people, and thus not representative of the entire population; moreover, the research—as can be seen in Figure 4—was mostly conducted in countries that can be considered WEIRD (i.e., Western, Educated, Industrialized, Rich, and Democratic; see Henrich et al., 2010 [96]—Translated: Western, Educated, Industrialized, Rich, and Democratic), i.e., countries that are highly developed and advanced technologically and industrially, whose population is mostly educated, and which possess democratic forms of government. It is hoped that future studies will be carried out with more homogeneous (representatively heterogeneous) samples in terms of socioeconomic characteristics, as well as adequately translating the instruments so that they can also be applicable in countries that do not reflect the WEIRD requirements described above—in fact, as already written, the instruments used for the most part by the studies proposed here are standardized and calibrated to populations that are considered WEIRD, and may not be suitable for application in other systems and cultures [83].

As a final characteristic of the samples, chosen by the authors of the studies, that future research could address is the quantity of participants: for most research in fact the samples are quite small, and this could further reduce the degree of representativeness. Studies on numerically larger samples could be conducted in the future.

On the other hand, with regard to the type of research conducted and the choices made by the authors of this research, it should be noted that none of the included studies are longitudinal in nature. It might be useful in the future to develop a study that follows a sample over a period of time, investigating the relationship between the Dark Triad and cognitive empathy, and finding, for example, if and how this relationship changes over the years—and then how it might be most effective to intervene depending on this. Finally, it would be desirable to use diversified sampling techniques, since in much of the research volunteer samples are used, and this could invite some biases into the study, as already specified.

Dealing, then, with the relationship between the Dark Triad and cognitive empathy, it might be helpful for future research to explore possible intervening variables of the relationship—this can be done by considering the Dark Triad as a whole or by breaking it down into its individual dimensions; to give an example, one could place alongside the tests used to assess the two constructs under consideration an instrument measuring the construct of the “motivation to lead”, defined as having an interest in being in charge of a group of people or a situation, which could make people with Dark Triad traits more prone to use manipulation strategies involving cognitive empathic skills.

The last aspect concerning future research is that of the instruments used in the assessment of the two constructs examined, many of which are self-report formats; this structure of the tests may bring with it some types of bias, for example, respondents may not answer truthfully. For future research, it would be optimal to use tests constructed in some other way or to pair these tests with instruments and/or scales that can reproduce the abilities that a person claims (as we have seen before, this is a factor of great importance in the assessment of the cognitive empathy skills of narcissists).

Turning to cognitive empathy, all instruments used to assess this are multidimensional, very often assessing both the cognitive part of empathy and the affective part—in fact, there is no one-dimensional test that assesses these two dimensions separately. The development of such an instrument could make the assessment of this ability more precise and effective, therefore give concrete support to the research done on this issue. Lastly, it was pointed out by Kajonius & Björkman [88] that, in the need to administer the designated English-language instrument to the test-taker (no translation being available for the language of the country in which the research takes place), it is not always possible to verify the participants’ English proficiency. This poses a major risk as far as the validity of the results found is concerned, and in the future, it is hoped that more translations of the tests and instruments will be produced, following current standardization norms, and that when the test is to be presented in English it will always be paired with an instrument or scale for measuring English proficiency.

## Figures and Tables

**Table 1:** Included studies that investigated the relationship between each Dark Triad trait and Cognitive Empathy as distinct traits.

Source	Sample Size	Narcissism r Value	Machiavellianism r Value	Psychopathy r Value
Wai & Tiliopoulos [45]	139	0.18	−0.08	−0.16
Jonason & Kroll [46]	516	−0.04	−0.05	−0.19
Jonason & Krause [48]	322	−0.14	−0.19	−0.23
Turner et al. [50]	1035	0.21	0.09	−0.06
Erickson & Sagarin [60]	532	−0.03	−0.11	−0.32
Vachon & Lynam [74]	369	−0.08	−0.23	−0.24
Pajevic et al. [78]	576	0.17	−0.16	−0.14
Fish [80]	136	−0.06	−0.19	−0.35
Schimmenti et al. [82]	799	0.20	−0.07	−0.23
Puthillamet al. [83]	212	−0.03	−0.15	−0.38
Gojković et al. [84]	263	0.24	0.13	−0.05
Wertag & Hanzec [85]	115	0.04	−0.03	−0.28
Bloxsom et al. [86]	262	0.05	−0.16	−0.31
Doyle [87]	267	0.15	−0.09	−0.21
Kajonius & Björkman [88]	278	−0.18	−0.23	−0.33
Quan et al. [89]	698	−0.01	−0.03	−0.09
Wertag et al. [90]	144	0.28	−0.08	−0.07
Kowalski et al. [91]	568	−0.31	−0.34	−0.35
Zirenko et al. [92]	690	0.03	−0.06	−0.12
Justice [93]	291	−0.17	−0.08	−0.20
Tobin [94]	153	0.12	0.05	0.20
Dinić et al. [95]	443	0.06	−0.00	−0.09

## Data Availability

Review data are already presented within the paper.

## Appendix A

**Table A1:** Key characteristics of the studies reviewed: reference, sample size, female percentage in the sample (gender distribution), mean age and age distribution, country, Dark Triad measure used in the study, Cognitive Empathy measure used in the study.

Reference	Sample Size	Female Percentage in the Sample (Gender Distribution)	Mean Age/Age Distribution	Country	Dark Triad Measure	Cognitive Empathy Measure
Wai & Tiliopoulos [45]	139	76.26%	M = 19.9, SD = 4.3 Range: // (Not given information)	Australia	- Mach-IV (Machiavellianism Scale-IV) [63] - NPI (Narcissistic Personality Inventory) [64] - LSRP (Levenson Self-Report Psychopathy Scale) [66]	EQ (Empathy quotient) [72]
Jonason & Kroll [46]	516	64.9%	M = 23.99, SD = 3.82 Range: 17–48	Germany	DDTD [62]	IRI [28,71]
Jonason & Krause [48]	322	75.16%	M (Mean) = 24.24, SD (Standard deviation) = 7.33 Range: 17–56	Australia	DTDD (Dark Triad Dirty Dozen) [95]	BES (Basic Empathy Scale) [73]
Turner et al. [50]	1035	66.09%	M = 19.66, SD = 3.36 Range: 18–53	USA	- SD3 [61] - NPI [64] - GNS (Grandiose Narcissism Scale) [67] - MACH-IV; [63] - MPS; (Machiavellian Personality Scale) [68] - LSRP [66] - SRP-III (Self-Report Psychopathy Scale-III)	- EQ [72] - BES [73] - HIFDS (How I Feel in Different Situations questionnaire) [29,76])
Erickson & Sagarin. [60]	532	53.20%	M_1_ = 40.43, SD_1_ = 12.62; M_2_ = 27.33, SD_2_ = 9.68 Range_1_: 18–75; Range_2_: 18–72	USA	DTDD [62]	IRI [28]
Vachon & Lynam [74]	369	44%	M, SD: // Range: //	USA	- SRP-III [65] - DTDD [62]	- ACME [74] - (Developmental version) - IRI [28] - BES [73]
Pajevic et al. [78]	576	56.60%	M = 32.91, SD = 10.94 Range: 18–68	Serbia	- SRP-SF (Self-Report Psychopathy Scale-Short Form) [109] - MACH-IV [63] - NPI [64]	- BES [73] - RMET (Reading the Mind in the Eyes) [72]
Fish [80]	136	83.09%	M = 26.51, SD = 12.32 “Range: 18–78	Australia	- SD3 [61] - PNI [70]	IRI [28]
Kaufman et al. [81]	670	52.5%	M = 36.07, SD = 11.82 Range: 19–74	USA	SD3 [59]	CASES (Cognitive, Affective, and Somatic Empathy Scales) [77]
Schimmenti et al. [82]	799	55%	M = 35.78, SD = 10.96 Range: 18–64	Italy	DTDD [62]	EQ [110]
Puthillam et al. [83]	212	70.75%	M = 21.70, SD = 3.41 Range: 18–33	USA	SD3 [61]	BES [73]
Gojković et al. [84]	263	73.11%	M = 18.3; SD= 1.65 Range: //	Serbia	SD3 [61] Serbian adaptation [95]	ACME (Affective and Cognitive Measure of Empathy) [74]
Wertag & Hanzec [85]	115	70.43%	M = 31.30, SD = 7.49 Range: 18–54	Croatia	SD3 (Short Dark Triad) [61]	- EQ−28 (Empathy quotient Short Form) [110] - IRI (Interpersonal Reactivity Index) [71]
Bloxsom et al. [86]	262	100%	M = 26.65, SD = 11.65 Range: 18–71	United Kingdom	SD3 [61]	QCAE (Questionnaire of Cognitive and Affective Empathy) [75]
Doyle [87]	267	79.4%	M = 20.59, SD = 5.40 Range: 18–52	Canada	SD3 [61]	EQ [72]
Kajonius & Björkman [88]	278	63%	M = 29.0, SD = 11.0 Range: 16–69	Sweden	SD3 [61]	IRI [28]
Quan et al. [89]	698	65.33%	M = 24.16, SD = 2.1 Range: //	China	SD3 [61]; Chinese version: [89])	IRI ([28] Chinese version; [111])
Wertag et al. [90]	144	57%	M = 22.18, SD = 2.26 Range: //	Croatia	SD3 [61]	ACME [74]
Kowalski et al. [91]	568	59.86%	M = 23.57, SD = 2.55 Range: 18–30	Italy	DTDD [62,82]	IRI [28]
Zirenko et al. [92]	690 (Russian sample: 308, Azerbaijani sample: 352)	Percentage of females present in the Russian sample: 80%, Percentage of females present in the Azerbaijani sample: 74%	Russian sample: M = 32.3, SD = 11.71; Azerbaijani sample: M = 30.5 SD = 10.40 Range Russian sample: 18–80; Range Azerbaijani sample: 17–74	Russia	DTDD [62]	QCAE [75]
Justice, [93]	291	52.9%	M: 19 Range: 18–29	USA	DTDD [62]	IRI [28]
Tobin [94]	153	85.62%	M, SD: // Range: //	USA	SD3 [61]	BES-A (Basic Empathy Scale in Adults) [112]
Dinić et al. [95]	443	49.9%	M = 28.13, SD = 6.66 Range: 19–49	Serbia	DTDD [62] SD3 [61]	ACME [74]

## Appendix B

**Table A2:** Main characteristics of the studies reviewed: reference, statistical main results, main results, study limitations, risks of biases.

Reference	Statistical Main Results	Main Results	Study Limitations	Risks of Biases
Wai & Tiliopoulos [45]	Narc. × CE = 0.18 * Primary Psych. (Primary Psychopathy) × CE = −0.16 Secondary Psych. (Secondary Psychopathy) × CE = −0.10 Mach. × CE = −0.08 * *p* < 0.05, two tailed. ** *p* < 0.01; two tailed.	All of the dark triad personalities showed a negative relationship with global empathy. In particular, they showed significant deficits in affective empathy, while with cognitive empathy there were weak positive correlations. Individuals with high levels of the dark triad appear to exhibit an empathic profile that allows them to retain the ability to read and evaluate others’ emotions, and subsequently use this sensitive information to formulate strategies by which they can acquire what they want, while their lack of affective empathy may lead them to overlook or ignore the potential harm that may be inflicted on others in the process. Narcissism was found to be positively correlated with cognitive empathy.	- There is a gender disparity in the sample. - The results for psychopathy of the second type should be interpreted with caution given its low reliability (a = 0.61), since no scale items were found to be responsible for this reduction. - The results of the facial expression task were relatively weak, perhaps suggesting that the medium used was not strong enough to elicit a sufficient emotional response.	- Mean sample age equals 24.24 years with a standard deviation of 7.33. - Age range not indicated. - Sample composed of college students only.
Jonason & Kroll [46]	PT × Psych. = −0.19 ** PT × Mach. = −0.05 PT × Narc. = −0.04 Notes. * *p* < 0.05. ** *p* < 0.01.	Psychopathy was negatively correlated with Perspective Taking (i.e., a measure of cognitive empathy). This study recognizes the distinction between the ‘‘darker’’ and ‘‘lighter’’ traits of the Dark Triad. Although both traits (Machiavellianism and psychopathy) may be part of an exploitative social strategy (Jonason & Webster, 2012; Mealey, 1995), this study suggests the way in which each trait might take a different approach when attempting to take advantage of others. Depending on different goals, each trait may steer toward qualitatively different forms of empathy.	- The recently translated German version of the DTDD that this study used has been criticized for its brevity. - The measure translated into German for measuring empathy may not take into account situational influences on empathetic tendencies. - The sample fits the WEIRD (i.e., Western, Educated, Industrialized, Rich, and Democratic; see 2010 [93]) category.	- Mean age of the sample equals 23.99 years with a standard deviation of 3.82. - There is a gender disparity in the sample.
Jonason & Krause [48]	Narc. (Narcissism) × CE (Cognitive Empathy) = −0.14 * Psych. (Psychopathy) × CE = −0.23 ** Mach. (Machiavellianism) × CE = −0.19 ** DT (Dark Triad) × CE = −0.22 **	All of the traits of the Dark Triad are associated with lower levels of cognitive empathy. Psychopathy and Machiavellianism correlate with low levels of affective empathy in contrast to narcissism. This suggests that each of the traits is accompanied by unique emotional deficiencies, but psychopathy facilitates the greatest number of emotional deficiencies.	- The measures used to assess psychopathy and “external orientation” had internal consistencies lower than traditional standards of 0.70 - While a multidimensional measure of empathy is used in the study, there are also theoretical complications with the conceptualization of this construct. - There is a gender disparity in the sample.	- Mean sample age equals 19.9 years with a standard deviation of 4.3. - Age range not indicated. - Use of three different measures for Dark Triad assessment; all of the tests employed in the measurement of both Dark Triad and cognitive empathy are multidimensional in nature. This generally means that the construct the test measures is better represented, given the accuracy in reporting the various aspects of it; but a majority of items (open-ended/multiple-response questions etc. to which the person to whom the test is administered responds) encapsulated in multiple scales and subscales can invite other types of bias within the analysis, like risking strain to the respondent.
Turner et al. [50]	CE (EQ) × Narc. (SD3) = 0.21 CE (EQ) × Narc. (NPI) = 0.18 CE (EQ) × Narc. (GNS) = 0.20 CE (EQ) × Mach. (SD3) = 0.09 CE (EQ) × Mach. (MACH-IV) = −0.09 CE (EQ) × Mach. (MPS) = 0.09 CE (EQ) × Psych. (SD3) = −0.06 CE (EQ) × Psych. (LSRP) = −0.14 CE (EQ) × Psych. (SRP) = 0.06 CE (BES) × Narc. (SD3) = 0.00 CE (BES) × Narc. (NPI) = −0.08 CE (BES) × Narc. (GNS) = 0.00 CE (BES) × Mach. (SD3) = −0.07 CE (BES) × Mach. (MACH-IV) = −0.21 CE (BES) × Mach. (MPS) = −0.12 CE (BES) × Psych. (SD3) = −0.24 CE (BES) × Psych. (LSRP) = −0.33 CE (BES) × Psych. (SRP) = −0.20 CE (HIFDS) × Narc. (SD3) = 0.12 CE (HIFDS) × Narc. (NPI) = 0.09 CE (HIFDS) × Narc. (GNS) = 0.10 CE (HIFDS) × Mach. (SD3) = −0.01 CE (HIFDS) × Mach. (MACH-IV) = −0.17 CE (HIFDS) × Mach. (MPS) = −0.01 CE (HIFDS) × Psych. (SD3) = −0.13 CE (HIFDS) × Psych. (LSRP) = −0.19 CE (HIFDS) × Psych. (SRP) = −0.11	Unlike affective empathy, which was negatively correlated with all three Dark Triad traits, cognitive empathy was not correlated with psychopathy while it was positively correlated with narcissism and Machiavellianism (both of which have above-average levels of cognitive empathy). The links between empathy and the Dark Triad were negative for affective empathy but positive for cognitive empathy except for psychopathy. In addition, the results suggest that people with higher levels of narcissism and Machiavellianism have the strongest cognitive empathy skills.	- Use of self-report measures. - Inability in empathy may be necessary but not sufficient to produce the observed patterns of association linking empathy to Dark Triad traits, and a functional field approach is warranted to map these links.	- Mean age of the sample equals 19.66 years with a standard deviation of 3.36. - Sample composed of undergraduate Psychology students. - There is a gender disparity in the sample.
Erickson & Sagarin. [60]	CE × Mach. = −0.11 * CE × Psych. = −0.32 *** CE × Narc. = −0.03 PT × Mach. = −0.24 *** PT × Psych. = −0.38 *** PT × Narc. = −0.21 ***	The objective of hypothesis H2 was to test correlations between everyday sadism and cognitive empathy, affective empathy, Dark Triad traits, and personality traits in a sample practicing BDSM when the CAST physics instructions explicitly indicated consent or nonconsent. Correlations for cognitive empathy were significant for the nonconsent condition but not significant (although in the same direction) in the consent condition.	- There was convenience sampling for both the BDSM group and the comparison group. All responses collected from the BDSM group were obtained from participants in BDSM conferences. - The non-BDSM sample was drawn from a population of college students and MTurk workers (Amazon Mechanical Turk is a crowdsourcing Internet service that allows computer programmers to coordinate the use of human intelligence to perform tasks that computers cannot do). - Although the use of these two groups has greater generalizability when combined, they are likely to differ from the general U.S. population. - An additional limitation of the obtained sample is that most participants, especially in the BDSM sample, identified themselves as white (Caucasian).	- Non-randomized sampling technique, sample of volunteers.
Vachon & Lynam. [74]	SRP Psych. Tot. × CE IRI = −0.24 SRP Psych. Total × CE BES = −0.29 SRP Psych. Tot. × CE ACME = −0.11 DD Mach. × CE IRI = −0.23 DD Mach. × CE BES = −0.19 DD Mach. × CE ACME = −0.02 DD Narc. × CE IRI = −0.08 DD Narc. × CE BES = 0.05 DD Narc. × CE ACME = 0.11	Low empathy in the children’s literature is often associated with lack of emotionality (e.g., callous, unemotional traits), so it is possible that high empathy is an expression of emotionality. High affective empathy was associated with emotional stability rather than emotionality—since empathy is a desirable trait, correlations between high empathy scores and undesirable traits (e.g., aggression, psychopathology, etc.) may be explained by social desirability effects. However, the scores of all three ACME scores were not correlated with social desirability.	- University samples from a Midwestern university were used. - A possible limitation of all self-report measures of cognitive empathy is that they ask individuals to assess their ability to identify and understand emotions rather than directly test this ability. This may be a problem both desirability effects, but also because self-reports are not appropriate for assessing cognitive ability.	- Mean age, standard deviation and the age range of participants were not provided. - There is a gender disparity in the samples. - The selected sample can be described as WEIRD (i.e., Western, Educated, Industrialized, Rich, and Democratic; see [93].
Pajevic et al. [78]	CE × Narc. = 0.17 ** CE × Mach. = −0.16 ** CE × Psych. = −0.14 ** ** *p* < 0.01; two-tailed.	Although narcissism showed higher self-reported cognitive empathy, this was not confirmed by performance in the emotion recognition task, which showed a non-significant relationship. This suggests that narcissism is associated with greater confidence in one’s ability to infer emotions in others, but superior performance in the actual task was not found. However, since narcissism emerged as a negative predictor of affective empathy and was not related to emotion recognition disorder, it could be argued that narcissism is associated with an empathic profile that allows one to understand how others feel without experiencing emotional contagion, which could be advantageous in conducting an exploitative and manipulative interpersonal style.	- The internal consistency of the RMET was much lower than the usual standard of 0.70, although still within the most liberal standards [94]. - A measure of emotion recognition based only on photographs is used, which have inherently limited ecological validity, as they cannot capture the nuances of actual social interactions. - The sampling method used is non-probabilistic (Snowball Technique or Avalanche Sampling).	- Sample possibly not representative for the following reasons: a) Non-probabilistic sampling technique; b) Gender imbalance in the sample; c) Average age of the sample.
Fish 2018 [80]	Mach. × PT = −0.19 * Psych. × PT= −0.35 *** Grandiose Narc. (Grandiose Narcissism) × PT = −0.06 Vulnerable Narc. (Vulnerable Narcissism) × PT = −0.17 * Note. * *p* < 0.05, ** *p* < 0.01, *** *p* < 0.001.	Gender and sensitivity to the expressive behavior of others have emerged as positive predictors of emotional regard. In contrast to existing research reporting that females possess higher levels of empathy than males, the data reported by this study suggest that being male contributed to a higher consideration of users’ feelings. In addition, it is surprising that perspective taking and empathic control did not contribute significantly to the pattern, given that they are related to the basic construct of empathy.	- The stimulus response tasks, while allowing inference of causality, were based on an analog methodology. In real Facebook exchanges, users might respond differently. - The assertive and defensive items showed questionable and poor internal reliability (α = 0.34 and 0.65 respectively)	- There is a gender disparity in the sample.
Kaufman et al. [81]	DT × CE = −0.14 ** DT (Partial) × CE = 0.12 **	In the regression, affective empathy was found to be a strong independent negative predictor of the Dark Triad, while cognitive empathy was a slight but significant positive predictor and independent predictor of the Dark Triad.	- All participants were recruited from paid online survey platforms. - Redundancy of the construct is a problem for distinguishing and measuring its components.	- Sample possibly not representative.
Schimmenti et al. [82]	DTDD Tot. (Total) × EQ CE = −0.02 DTDD Mach. × EQ CE = −0.07 DTDD Psych. × EQ CE = −0.23 ** DTDD Narc. × EQ CE = 0.20 * * *p* < 0.05; ** *p* < 0.01 (two tails)	Dark Triad traits were significantly associated with reduced theory of mind, alexithymic traits and low empathy.	- Sample composed entirely of adult volunteers. - Possible social desirability bias. - The characteristic of being a cross-sectional study does not allow consideration of other variables that may be present in the relationship between Dark Triad and cognitive empathy.	- Sampling bias due to non-probability sampling technique (volunteer sample).
Puthillam et al. [83]	CE × Mach. (SD3) = −0.15 CE × Narc. (SD3) = −0.03 CE × Psych. (SD3) = −0.38 *** CE × DT (SD3) = −0.24 *** **p* ≤ 0.05, ** *p* ≤ 0.01, *** *p* ≤ 0.001	The Dark Triad negatively predicts cognitive empathy, especially with regard to psychopathy. Machiavellianism was a significant negative predictor only of affective empathy, while narcissism predicted neither. Moreover, Machiavellian individuals might use cognitive empathy to charm and manipulate others ([43]), while a low level of affective empathy might facilitate this exploitation.	- Because the GERT-S measures emotion recognition only across Caucasian actors, it is possible that people of non-Caucasian origin are not as skilled at recognizing emotions looking at interactions between Caucasian people. This is especially important to note because 65.09% of the participants were Indian. - The social desirability scale showed moderate internal consistency in the current sample (α = 0.58), making it questionable. Similarly, the alpha for psychopathy was also slightly lower (α = 0.68) than the acceptable level. - There is a gender disparity in the sample. - Since, theoretically, the Dark Triad consists of overlapping traits, interpretive doubts accompany the resulting shared statistical variances.	- Mean age of the sample equals 21.70 years with a standard deviation of 3.41.
Gojković et al. [84]	CE × Mach. = 0.13 * (*p* = 0.040) CE × Narc. = 0.24 *** CE × Psych. = −0.05 CE × DT Tot.= 0.44 *** Notes: * *p* <0.05; ***p* <0.01; *** *p* <0.001;	The close connections of Machiavellianism and cognitive empathy with the SD3N-admiration axis speak to the manipulative and duplicative quality of this cluster of four traits. There is a direct link between the SD3N-admiration nodes and cognitive empathy, but with none of the indices of affective empathy. In narcissists, the presence of cognitive empathy is primarily indicative of an instrumentally refined ability to read the emotional states of others. Thus, both narcissism and Machiavellianism mask their fundamentally aversive character, as the absence of an affective response unequivocally resonates the antagonistic nature of SD3N. In their work, Vachon and Lynam [79] report that cognitive empathy is poorly associated with externalizing psychopathology.	- This study was based on self-report instruments from a relatively small, nonclinical, and unrepresentative sample of adolescents from a geographically limited area, which potentially limits the variability of responses and the power of statistical analyses. - The study was based on the validated Serbian version of SD3 [62] and the unvalidated translations of the NARQ and ACME. Although both the NARQ and SD3 have been used in studies including adolescents, they were originally developed and validated on adult respondents. - An additional limitation is due to the relatively modest reliability of the SD3N.	- There is a gender disparity in the sample. - The mean age of the sample is extremely low, equal to 18.3 years with a standard deviation of 1.65. - Age range not indicated.
Wertag & Hanzec [85]	EQ28 × Mach. = −0.155 CE × Mach. = −0.030 PT30 ^×^ Mach. = −0.372 ** EQ28 × Narc. = 0.000 CE × Narc. = 0.049 PT × Narc. = −0.216 * EQ28 × Psych. = −0.410 ** CE × Psych. = −0.285 ** PT × Psych. = −0.477 ** * *p* < 0.05, ** *p* < 0.01	The correlations between EQ−28 and the Dark Triad are low/moderate and negative in nature.	- There is a gender disparity in the sample. - The sample considered is considered unrepresentative because of its size. - There are not ideal rates of internal consistency for all scales, especially for short scales.	- The ultimate goal of this study is not to evaluate the relationship between the desired constructs.
Bloxsom et al. [86]	CE × Mach. = −0.161 ** CE × Narc. = 0.058 CE × Psych. = −0.310 *** Note: * *p* < 0.05, ** *p* < 0.01, *** *p* < 0.001.	Cognitive empathy is negatively correlated with Machiavellianism and psychopathy, while affective empathy has been associated negatively with all DT traits. There is an assumption that participants self-report truthfully; however, dark personalities are prone to dishonesty and manipulation; in the case of grandiose narcissism, one’s abilities are exaggerated, which may have led to the positive relationship with cognitive empathy. Indeed, the SD3 measures grandiose narcissism (rather than vulnerable narcissism), and, as a result, participants with higher levels of this trait may have overestimated their ability to understand others or their relational security.	- The sample consisted entirely of women (predominantly Caucasian British students), and thus the pattern established here refers only to this demographic group. - This study used only self-report assessments, which require participants to have sufficient metacognitive awareness to make an accurate self-assessment.	- Sample consisting entirely of women, mostly Caucasian students and falling into the WEIRD category (i.e., Western, Educated, Industrialized, Rich, and Democratic; see [93]). - Mean age of the sample equals 26.65 years with a standard deviation of 11.65.
Doyle [87]	CE × Mach. = −0.09 CE × Narc. = 0.15 * CE × Psych. = −0.21 ** Note: *p* < 0.10. * *p* < 0.05. ** *p* < 0.01. *** *p* < 0.001.	Machiavellianism has not been significantly associated with cognitive empathy. It is possible that individuals with higher Machiavellianism scores simply possess “normal” abilities in these domains. Narcissism, on the other hand, was found to be positively associated with cognitive empathy. The ability to assume another person’s perspective would facilitate attempts to manipulate and deceive others ([45,48];). However, it is possible that individuals with higher narcissism tend to “over-report” their cognitive empathic abilities. Because the EQ is a self-report measure, individuals with higher narcissism scores may have reported an overly favorable view of their empathic abilities. Psychopathy was negatively associated with cognitive empathy. This finding suggests that individuals with high psychopathy scores are unable to lie effectively [48,95]. It could be argued that psychopaths may have earned a reputation as liars and manipulators because of the frequency with which they enact these behaviors. However, there is only mixed evidence to support the idea that psychopathy is associated with success in these endeavors [96,97,98].	- There is a gender disparity in the sample. - The proposed sample comes from a university setting typical of a WEIRD society (i.e., Western, Educated, Industrialized, Rich, and Democratic; cf. [93]).	- Mean age of the sample equals 20.59 years with a standard deviation of 5.40.
Kajonius & Björkman. [88]	CE (IRI PT) × Mach. = −0.23 CE (IRI PT) × Narc. = −0.18 CE (IRI PT) × Psych. = −0.33 Note. r > 0.10 was significant at *p* < 0.05 and r > 0.16 at *p* < 0.01.	The results showed that it is more the lack of empathic disposition rather than inability that characterizes dark personalities. First, the Dark Triad had a very strong relationship with trait-based empathy. Second, the Dark Triad had a weak (almost nonexistent) relationship with ability-based empathy. Third, cognitive ability explained most of the ability-based empathy. This study, by testing negative relationships with the IRI scales, confirms the general notion that the Dark Triad and dispositions to empathy are negatively correlated [40].	- Results obtained through self-report measures. - No control could be performed on study participants and their English proficiency levels it was only a requirement that they had to report being fluent in order to participate in the survey. - The sample was strongly characterized by college-educated individuals associated with interests in human resources via the online LinkedIn platform, most of whom were female, not known for high Dark Triad scores.	- The English language skills of the sample are unknown. - Unbalanced sample in terms of age, gender, and sampling.
Quan et al. [89]	CE (IRI PT) × Mach. = −0.036 CE (IRI PT) × Narc. = −0.012 CE (IRI PT) × Psych. = −0.090 * Note: * *p* < 0.05, ** *p* < 0.01	The Machiavellianism score is significantly negatively correlated with the Empathic Concern and Perspective Taking scores. The narcissism score is not significantly correlated with the empathic subscales.	- Sample mainly composed of Chinese college students.	- There is a gender disparity in the sample. - The mean age of the sample is extremely low, equal to 24.16 years with a standard deviation of 2.1. - Age range not indicated.
Wertag et al. [90]	CE × Mach. = −0.08 CE × Narc. = 0.28 ** CE × Psych. = −0.07 Notes: * *p* < 0.05. ** *p* < 0.01.	Regarding the relationships of the dark traits with empathy, narcissism was found to be positively associated with cognitive empathy, while the other dark traits were associated with lack of affective empathy, consistent with the notion of narcissism as a “brighter” trait.	- Relatively small sample.	- Sample composed entirely of college students, thus unrepresentative. - Mean sample age equal to 22.18 years with a standard deviation of 2.26. - Age range not indicated.
Kowalski et al. [91]	IRI PT × DTDD Psych. = −0.35 * IRI PT × DTDD Mach. = −0.34 * IRI PT × DDDT Narc. = −0.31 * Note. Bonferroni correction applied to correlations (significant at *p* < 0.004). *p* < 0.05. *p* < 0.001.	None of the dark tetrad traits were found to significantly correlate with the “negative affect”.	- The sample used in this study consists largely of young people with above-average education. - Given the results of this study, it is possible that IRI confuses empathy with sympathy, leading to a mismeasurement of cognitive empathy [56].	- There is a gender disparity in the sample. - Mean age of the sample equals 23.57 years with a standard deviation of 2.55.
Zirenko et al. [92]	Mach. × PT = 0.09 Psych. × PT = 0.07 Narc. × PT = 0.09 Mach. × CE = −0.06 Psych. × CE = −0.12 Narc. × CE = 0.03 Note. * *p* < 0.05, ** *p* < 0.01, *** *p* < 0.001	In the Azerbaijani sample, ITE showed positive associations with online simulation and cognitive empathy, and negative associations with emotion contagion. Risk propensity was positively associated with Machiavellianism and Perspective Taking in both samples, particularly with proximal responsiveness and cognitive empathy in Azerbaijan, with affective empathy and narcissism in Russia, and negatively with rationality and Emotion Contagion in both samples. In Russia and Azerbaijan, similar relationships were observed between empathy and risk propensity, rationality, and DT traits. Previous cross-cultural studies have shown that DT traits and emotional intelligence are not correlated, therefore, the observed relationship between empathy and DT traits is probably not mediated by emotional intelligence, which was not measured in this study.	- The results of this study could be influenced by the different severities of the COVID−19 epidemic in the two countries. In addition, it was noted how strict normative regulation of behavior makes it almost impossible to reveal personality regulation of choices. - The IT of emotions and subscales of cognitive empathy emerged as the variables predicting caring for others versus caring for self as the reason for wearing a surgical mask, suggesting that these components of emotional regulation play a role in prosocial behavior. - We also need to study more deeply why, compared with Russia, collectivist countries showed higher indices of cognitive, but not emotional empathy, which might be expected in cultures with closer interpersonal relationships.	- Study based on public behavior given the epidemic of COVID−19 in two countries and the influences that certain personality types or disorders have on the implementation of these. Therefore, the ultimate purpose of this research is not to measure the relationship between the Dark Triad and cognitive empathy. - There is a gender disparity in the samples. - Cross-cultural research.
Justice, [93]	Mach. × PT = −0.08 Psych. × PT = −0.20 * Narc. × PT = −0.17 ** *p* < 0.01	As expected, many of the Dark Tetrad traits correlated negatively with empathy. Machiavellianism correlated negatively with Empathic Concern and the Empathy subscale of the I7 scale. Psychopathy also correlated negatively with these two scales (Empathic Concern and I7 Empathy subscale), as well as correlated negatively with Perspective Taking. Narcissism correlates positively with some of the empathy scales; the only negative correlation found was between narcissism and Perspective Taking. As expected, psychopathy was significantly predictive of low empathic concern and predictive of low empathy on the I7. Narcissism resulted in positive predictions on all but one subscale (Perspective Taking). Machiavellianism showed no indication of being predictive of decreased empathy.	- The sample of participants was taken from a private, Christian-affiliated university. It is assumed that people from religious backgrounds were mostly present, as well as having higher levels of socioeconomic status. - The presence of an inaccessible sample of inmates. The accessible sample was not of inmates, was age-limited, and from similar backgrounds, thus less likely to have a high level of dark tetrad traits. - The sample included a disproportionate number of Caucasian individuals and more females than males.	- Mean sample age equal to 19 years with standard deviation not shown. - Very limited context from which the sample was taken.
Tobin, [94]	Mach. × CE = 0.056 Narc. × CE = 0.123 Psych. × CE = 0.204 * * Correlation is significant at the 0.05 level.	It is hypothesized that cognitive empathy and empathic ability are predictive of infrahumanization and that the Dark Triad traits are not predictive of it. No significant regression equations were found.	- Psychology has not yet created a conclusive theory of emotions, and to date there are many different theories in this area.	- This study does not aim to highlight the relationship between the Dark Triad and cognitive empathy. - There is a gender disparity in the samples. - Mean age, standard deviation of this and the age range of the participants was not indicated.
Dinić et al. [95]	CE × DTDD Mach. = −0.00 CE × DTDD Psych. = −0.09 CE × DTDD Narc. = 0.06 CE × SD3 Mach. = 0.02 CE × SD3 Psych. = −0.15 ** CE × SD3 Narc. = 0.04 ** p* < 0.05.	The dark traits of each scale, especially psychopathy, are not related to affective empathy, but when it comes to cognitive empathy a correlation is found.	- Only self-report measures were used. - The data were cross-sectional in nature. - The samples used are of modest size. - A limited nomological network was evaluated.	- This study adapts a measure to the Serbian language; its ultimate goal therefore is not the assessment of the relationship between the Dark Triad and cognitive empathy.

Note: * *p* < 0.05. ** *p* < 0.01. *** *p* < 0.001.
